# Gender differences in pre-surgical hip arthroplasty patients

**DOI:** 10.3389/fgwh.2025.1565899

**Published:** 2025-04-22

**Authors:** Elisabet Ripoll-Romero, Zaida Agüera, Montserrat Puig-Llobet, Marta Bassas, Maria Cristina Manzanares-Céspedes, Jordi Galimany-Masclans

**Affiliations:** ^1^ Department of Orthopedic Surgery, Hospital Clinic de Barcelona, Barcelona, Spain; ^2^ Departament D’Infermeria de Salut Pública, Salut Mental I Maternoinfantil, Facultat D’Infermeria, Universitat de Barcelona, Barcelona, Spain; ^3^ Research Group in Mental Health, Psychosocial and Complex Nursing Care (NURSEARCH), Facultat D’Infermeria, Universitat de Barcelona, Barcelona, Spain; ^4^ Psychoneurobiology of Eating and Addictive Behaviors Group, Neurosciences Programme, Bellvitge Biomedical Research Institute (IDIBELL), Barcelona, Spain; ^5^ CIBER Fisiopatología Obesidad y Nutrición (CIBERobn), Instituto de Salud Carlos III, Madrid, Spain; ^6^ Human Anatomy and Embryology Unit, Department of Pathology and Experimental Therapy, Faculty of Medicine and Health Sciences, University of Barcelona, Barcelona, Spain

**Keywords:** hip arthroplasty, anxiety, depression, quality of life, gender

## Abstract

**Introduction:**

There is growing evidence that gender may influence surgical outcomes, rehabilitation, and overall patient experience in various orthopedic procedures. The main objective of the present study was to explore gender-based differences in patients undergoing hip arthroplasty surgery to understand better how they might impact the preoperative experience and contribute to personalized patient care.

**Methods:**

A cross-sectional study was conducted with *n* = 180 pre-surgical participants (*n* = 84 females, *n* = 96 males). All patients were consecutively admitted for primary hip arthroplasty surgery at the orthopedics surgery and traumatology unit of the Hospital Clínic of Barcelona (Spain), between October 1, 2023 and July 31, 2024. The assessment consisted of a socio-demographic form and two questionnaires: the Hospital Anxiety and Depression Scale (HADS) and the 5-level EQ-5D version (EQ-5D-5l).

**Results:**

Both groups consisted of a similar percentage of males and females. Females presented statistically significantly higher levels of preoperative anxiety than males (*p* = .050), but no significant differences appeared in the depression subscale (*p* = .382). Regarding quality of life, females reported significantly higher scores on difficulties in mobility (*p* = .037), usual activities (*p* = .027), and perceived pain (*p* = .004) compared to males. Finally, greater anxiety and depression scores were associated with poor self-perceived health in males but not in females.

**Conclusions:**

This study is essential to bridge gaps in knowledge regarding gender differences in patients undergoing hip arthroplasty. Clinically, these results underline the importance of considering gender-specific factors when planning interventions and support for hip arthroplasty patients to improve outcomes and overall well-being.

## 1 Introduction

Hip arthroplasty is one of the most effective surgical interventions for improving the quality of life in patients suffering from severe hip joint conditions, such as osteoarthritis, hip fractures, congenital dysplasia, or avascular necrosis. While significant advancements have been made in surgical techniques, patient outcomes, and rehabilitation, there is growing evidence suggesting that preoperative factors, including physical, psychological, and social aspects between patients of both sex o genders, potentially influence outcomes after surgery (1, 2).

There are well-documented anatomical and biological sex-based differences between men and women, which can influence the manifestation and progression of joint diseases. For example, studies have shown that women tend to develop osteoarthritis later than men, but with more rapid disease progression and higher levels of disability before surgery. Additionally, differences in bone density, muscle mass, and biomechanics between sex could impact pain perception, joint deterioration, and surgical outcomes (3).

Research suggests that women and men often perceive and report pain differently, with women generally reporting higher levels of pain and disability before surgery (4, 5). Comorbidities such as obesity, diabetes, cardiovascular diseases, and osteoporosis tend to present differently between men and women (6) as well as psychopathological conditions such as anxiety and depression, with women reporting higher obesity, levels of pain and functional limitations than men (7). As a consequence of reporting a high level of pain, women also use more non-prescription and prescription analgesic medications than men (8).

Preoperative anxiety is a significant concern in surgical setting, affecting a large proportion of patients worldwide. The global prevalence of preoperative anxiety among surgical patients are approximately 48%, with higher rates in Africa and Asia (9). In a study of cardiac surgery patients, 94% experienced preoperative anxiety (10). Psychological and social factors, including levels of depression, anxiety, and social support have been found to influence patients' recovery trajectories after hip arthroplasty. In this sense, gender-based differences have been described in the literature, with women being more likely to experience higher rates of preoperative anxiety and depression, which may affect their perception of pain and overall satisfaction with surgery (11). On the other hand, other studies suggest the opposite direction of this relationship, indicating that individuals living with chronic pain are also more vulnerable to developing higher levels of psychopathology. Also, it should be noteworthy that recent studies suggest a bidirectional pathway between pain and anxiety (12) Sociocultural expectations have been identified as relevant factors influencing pain and emotional expression in men (13). Additionally, gender roles may influence how patients engage with postoperative rehabilitation, impacting functional outcomes (14).

Although the literature extensively addresses sex differences in hip arthroplasty outcomes, limited attention has been given to how gender roles and psychosocial factors such as anxiety, depression, pain perception, and quality of life interact in women undergoing this procedure. In addition, to our knowledge, scarce literature so far has addressed gender-based differences in presurgical quality of life in this clinical population. Given the increasing focus on personalized and equitable healthcare, it is crucial to fill this gap by providing a comprehensive analysis of these psychosocial variables in a population frequently underrepresented in clinical research. Therefore, this study aimed to explore these gender-based differences to understand better how they might impact preoperative experience and contribute to personalized patient care.

## 2 Material and methods

We conducted a cross-sectional cohort study. Ethics approval was received from the Ethical Committee at the Hospital Clínic of Barcelona (ref HCB/2023/0015). All participants signed the informed consent.

### 2.1 Participants

Adults who underwent primary hip arthroplasty at our university Hospital Clínic of Barcelona (Spain) between 1 October 2023 and 30 June 2024 were contacted to participate in this study. The inclusion criteria consisted of all patients who were consecutively admitted for hip arthroplasty in the period indicated above. Exclusion criteria consisted of revision arthroplasty and admission via the emergency department for a fracture requiring arthroplasty. Patients who did not complete the entire battery of questionnaires were also excluded from the analyses.

### 2.2 Data collection

The following sociodemographic, clinical, and psychopathological data were collected for each participant: age, gender, coexistence, academic level, psychiatric problems, and use of psychopharmacological treatment. The levels of pre-surgical anxiety and depression were assessed using the validated Spanish version of the Hospital Anxiety and Depression Scale (HADS) (15). In addition, self-perceived quality of life was assessed using the Spanish version of the 5-level EuroQol (EQ-5D-5l) (16).

### 2.3 Statistical analysis

Statistical analyses were carried out with SPSS v.29 for Windows. First, we conducted a Kolmogorov–Smirnov normality test to check if there was a normal distribution and to determine which statistic to use. Sociodemographic and clinical characteristics were compared between genders using a *t*-test and Chi-square test for continuous and categorical variables, respectively. Additionally, because *p*-values are highly dependent on sample sizes, we included Cohen's d coefficient to estimate the effect sizes between two mean differences. The effect size of the mean differences was considered medium for |*d*| > 0.5 and large for |*d*| > 0.8. The correlations were estimated using Pearson's correlation coefficient.

## 3 Results

### 3.1 Description of the study sample

A total of 247 patients were recruited from October 2023 to June 2024. The final sample comprised 180 participants who met the inclusion criteria. Patients who had undergone bilateral arthroplasty (*n* = 11) and those with incomplete data in the questionnaires (*n* = 56) were excluded from the analyses.

The distribution of males and females in our sample was similar (53.33%, and 46.66%, respectively). The mean age of the patients was 65.9 years (SD 11.6), with females presenting a mean age of 66.38 (SD = 11.75) years and men 65.65 (SD = 11.57) years. No significant age differences were found between the gender-based groups (*t* = 422; *p* = .337). Most of the participants lived with first-degrade families, both females and males. Males reported non-significantly more high school and university studies than females, while females reported more primary education. Females reported significantly more psychological and sleep problems, as well as more psychopharmacological treatment than males (see Table 1).

**Table 1:** Sociodemographic characteristics of the sample.

Sociodemographic variables	Total		Females		Males		*Χ* ^2^
	*n* = 180	%	*n* = 84	%	*n* = 96	%	
Family cohabitation							.676
Alone	34	18.9	18	10	16	8.9	
1st grade	134	74.4	60	33.3	74	41.1	
Others	12	6.7	6	3.3	6	3.3	
Studies							.115
Primary	47	26.1	28	15.6	19	10.6	
Secondary	66	36.7	27	15	39	21.7	
University studies	67	37.2	29	16.1	38	21.1	
Psychiatric problems							.022
Yes	38	21.1	24	13.3	14	7.8	
No	142	78.9	60	33.3	82	45.6	
Sleep problems							.042
Yes	12	6.7	9	5	3	1.7	
No	168	93.3	75	41.7	93	51.7	
Psychopharmacological treatment							.008
Yes	42	23.5	27	15.1	15	8.4	
No	137	76.5	56	31.3	81	45.3	

Significance *p* < .05.

### 3.2 Psychopathology and quality of life

Table 2 shows the comparison between the two groups differentiated by gender. Regarding psychopathology measured by the HADS, females presented statistically significantly higher levels of preoperative anxiety than males (*p* = .050, |*d*| = .247), but no significant differences appeared in the depression subscale (*p* = .382, |*d*| = .45).

**Table 2:** Comparison between gender on pre-surgical anxiety and depression (HADS) and quality of life (EQ-5D-5l).

Psychopathological and quality of life variables	Total		Females		Males		*p*	*|d|*
	*n* *=* 180		*n* *=* 84		*n* *=* 96			
	Mean	SD	Mean	SD	Mean	SD		
HADS								
HADS-A	6.64	4.205	7.19	4.216	6.16	4.158	.**050**^a^	.247
HADS-D	5.96	4.083	6.06	3.730	5.88	4.387	.382	**.045** ^†^
EQ-5D-5l								
Mobility	3.41	0.830	3.52	0.799	3.30	0.848	.**037**^a^	.269
Self-care	2.73	1.034	2.82	1.088	2.66	0.982	.143	.160
Usual activities	3.42	1.008	3.57	0.985	3.28	1.013	.**027**^a^	.290
Pain/discomfort	3.73	0.790	3.89	0.761	3.58	0.790	.**004**^a^	.399
Anxiety/depression	2.41	1.263	2.56	1.320	2.27	1.201	.063	.229
Self-perceived health	49.84	20.75	48.42	21.83	51.09	19.80	.195	.129

SD, standard deviation; HADS, hospital anxiety and depression scale; HADS-A, anxiety scores; HADS-D, depression score; EQ-5D-5l, EuroQol-5 dimensions-5 levels.

^a^ Bold: significant parameter.

^†^Bold: effect size into the range mild-moderate (|*d*| > 0.50) to large-high (|*d*|>0.80).

Regard to quality of life measured by EQ-5D-5l, the results showed that females reported significantly higher scores on difficulties in mobility (*p* = .037, |*d*| = .269), usual activities (*p* = .027, |*d*| = .290), and perceived pain (*p* = .004, |*d*| = .399) compared to males. Non statistically gender-based differences were found in self-care (*p* = .143, |*d*| = .160) and anxiety and/or depression self-reported scores (*p* = .063, |*d*| = .229), neither in the visual analogic scale of self-perceived health (scoring 0–100) (*p* = .195, |*d*| = .129).

Table 3 displays the correlations between anxiety, depression (measured by HADS), and quality of life (by EQ-5D-5l) separately for each of the gender-based groups to identify possible gender-specific clinical characteristics. Among female participants, significant positive correlations emerged between anxiety and depression (HADS) and anxiety/depression (EQ-5D-5l). Specifically, anxiety showed a correlation of *r* = 0.418 (*p* = 0.5), while depression correlated at *r* = 0.484 (*p* = .01).

**Table 3:** Correlations of Pearson between anxiety and depression (HADS) and quality of life (EQ-5D-5l).

Gender groups	HADS	Mobility	Self-care	Usual activities	Pain discomfort	Anxiety/depression	Self-perceived health
Females	HADS-A	−.085	.068	.043	.218	.418*	−.014
	HADS-D	.016	.109	.175	.212	.484**	−.188
Males	HADS-A	.275	.052	.196	.245	.341	−.417*
	HADS-D	.358	.245	.326	.220	.415*	−.587**

HADS, hospital anxiety and depression scale; HADS-A, anxiety scores; HADS-D, depression score.

* *p* < .05 **p < .001.

In male participants, significant positive correlations were observed between depression, as measured by the HADS and the anxiety/depression (EQ-5D-5l) (*r* = .415, *p* = .05), though no such correlation was found for anxiety (HADS). Additionally, negative correlations were identified between anxiety (HADS), depression (HADS), and self-perceived health (VAS of the EQ-5D-5l) (*r* = −0.417, *p* = .05). A strong negative correlation was noted between depression (HADS) and self-perceived health (VAS of the EQ-5D-5l (*r* = −0.587, *p* = .01). Non statistical correlations were found between the other subscales.

## 4 Discussion

The present study attempted to address a relevant issue in patients undergoing hip arthroplasty. It aimed to provide insights into gender-based differences in these patients by comparing levels of anxiety and depression, as well as quality of life before surgery to design differential and tailored cures and interventions based on sex or gender. The main findings suggest gender-based differences in anxiety symptomatology, problems in mobility, impaired usual activities, and pain-discomfort, with females being who reported higher scores in all these variables. In addition, only in the male group, greater anxiety and depression were associated with poor self-perceived health, but not in females.

Regarding psychopathology, our results are in line with previous studies that have observed higher levels of preoperative anxiety in females compared to males (17–19). This observation is supported by previous literature reporting physiological and psychological gender-based differences in response to anxiety (20–22). Previous studies have suggested that factors such as genetics, neural and physiological functions of the brain as well as hormonal variations may contribute to higher levels of anxiety and depression in women compared to men (23–25). This finding is noteworthy, as the tendency for women to experience higher level of anxiety might influence their perception of the surgical process and recovery, though further research is needed to explore the exact nature of the relationships (26, 27).

Our findings also showed quality of life gender-based differences. A trend was observed suggesting that females undergoing hip arthroplasty reported greater mobility limitations and more significant impairments in daily activities compared to males of the same age and in the same conditions. These gender disparities could be associated with several factors. First, the higher prevalence of osteoporosis in women, particularly postmenopausal, leads to reduced bone density and increases the risk of hip fractures or joint degeneration (28). Second, females generally have lower muscle mass and strength than men, which can affect their ability to maintain stability and functional mobility, especially in the presence of a degenerative joint (29). Third, higher reported pain levels in females could potentially be related to greater physical and psychological limitations in performing daily activities (30). Fourth, gender can play a significant role in how patients access and engage with healthcare services. Females are more likely to delay seeking medical care for joint-related issues due to caregiving roles, which can result in more advanced stages of joint damage and functional decline by the time they undergo surgery (31). In contrast, males might seek treatment earlier but may not adequately express their concerns or fears (32). Finally, these gender differences may be also explained by sociocultural factors. That is, women may face additional barriers to physical activity, such as caregiving responsibilities, which can further affect their mobility and ability to perform daily tasks.

Concerning pain perception, our results are consistent with previous studies indicating that women tend to report higher levels of pain and disability prior to surgery (4, 5). The literature suggests that women may experience higher pain sensitivity and lower pain tolerance, which could be related to differences in hormonal and inflammatory milieu, central pain processing, body composition, or other biological, psychosocial, and cultural factors (13). Sex hormones could influence pain perception; variations and decreases in estrogen levels have also been reported to be associated with a lower pain threshold than in men and a higher level of nociception (33, 34). A study conducted in 2023 demonstrated that women are influenced by hormones, as pain perception varies according to the menstrual cycle and relates a lower level of pain tolerance to lower blood estrogen levels (33). In our study, no significant differences were found between pain and anxiety, neither in women nor in men. This surprising finding, although surprising, is not consistent with previous studies reporting that when a person has higher levels of anxiety, he or she also has higher levels of pain (13, 35). However, the lack of association observed in our study could be linked to the fact that the patients were receiving controlled analgesia. Furthermore, the anxiety detected in this case seems to be of a “non-psychopathological” nature, associated with the pre-surgical process. All these contributions support the importance of recognizing and assessing the psychological and emotional well-being of these patients, in a gender-differential manner, to provide quality nursing care adapted to the needs of each patient, considering gender-differential components.

### 4.1 Limitations and strengths

The current study presents some limitations that should be considered. Firstly, the study design does not allow us to differentiate between patients with a diagnosis of affective mental disorder and the subclinical symptomatology related to the pre-surgical process itself. However, it should be noted that the scores of the sample do not reveal alertness for serious mental problems. Second, At the time of collection, we do not know how long they have been in pain. We do not know if a long period of pain can influence the surgical process.

Further studies should control these limitations, screening the sample by mental disorders and considering other variables of interest such as quality of life perception, and satisfaction in the surgical process. Third, the study's design limits our ability to establish a direct causal link between surgery and the observed outcomes in anxiety, depression, and quality of life. To address this limitation, future research should employ controlled experimental designs, which would allow for a more precise determination of the surgery's impact on these psychological and well-being measures. Despite its limitations, this study has certain strengths that should be highlighted. Most studies on hip arthroplasty so far have focused on psychosocial factors that may influence the surgery outcome and prognosis of these patients. However, there was a lack of studies such as this one that analyzed gender differences to identify differential profiles that may require specific nursing care. Our findings highlight the need for a gender-sensitive approach to preoperative assessment and postoperative care to address the gender-based challenges faced by females and males undergoing hip arthroplasty.

### 4.2 Implications for clinical practice

The findings might provide the basis for evidence-based, personalized interventions that address differential needs based on sex and gender, ultimately improving outcomes and reducing health inequalities. Previous literature provides evidence that preoperative assessments need to be tailored to account for sex-based anatomic differences to select the most appropriate surgical techniques and implants. But additionally, our results support the need to educate patients about potential differences in recovery and outcomes based on gender, which may help set realistic expectations and improve patient satisfaction. Finally, addressing psychological and emotional needs based on gender may improve overall patient satisfaction and long-term outcomes, as females may need more support in managing postoperative pain and functional limitations, while males would need to work more on self-perceived health when presenting with anxiety or depressive symptoms.

## 5 Conclusions

In conclusion, our findings underscore the importance of considering specific factors, not only anatomical and hormonal factors based on sex, but also gender-related factors, such as affective psychosocial aspects and perceived quality of life, in the preoperative and postoperative management of hip arthroplasty patients.

## Data Availability

The original contributions presented in the study are included in the article/Supplementary Material, further inquiries can be directed to the corresponding authors.
